# Physiological Temperance Society

**Published:** 1842-01

**Authors:** 


### PHYSIOLOGICAL TEMPERANCE SOCIETY.

A society has been organized in the Medical Institute of Louisville, under the above title. Its object is to investigate the whole subject of alcoholic intemperance, together with the abuse of other stimulants and narcotics. As far as we know, over stimulation has not before been made a *speciality*, though from its prevalence and desolating effects, it well deserves such consideration. The society, which was organized as late as the 23d ult., already embraces more than a hundred members. Its meetings are semi-monthly; from the first of November to the first of March, in the hall of the Institute; where papers will be read and discussions carried on, relative to the causes, pathological effects and therapeutic means, both prophylactic and curative, of intemperate stimulation with intoxicating beverages and other excitants. The society respectfully invites communications on all the topics lying within its purview; and especially autopsic reports on those who have died from intemperance. The officers for the present year are *President*, Dr. Daniel Drake; *Vice-Presidents*, Dr. Th. J. Kennedy, of Tenn., Mr. C. J. Clark, of Ala., Mr. H. C. Holmes, of Mi., and Mr. E. R. Roe, of Ia.; *Secretary*, Mr. T. Bohannan; and *Treasurer*, Mr. J. F. Bull, both of Louisville.

